# The neuro-psychological manifestations of COVID-19 in healthcareworkers

**DOI:** 10.1192/j.eurpsy.2024.334

**Published:** 2024-08-27

**Authors:** A. Ghenim, I. Kacem, A. Chouchane, A. Aloui, C. Sridi, A. Fekih, M. Hafsia, M. Maoua, M. Kahloul, N. Mrizak

**Affiliations:** ^1^Occupational Medicine Department, Farhat Hached Academic hospital; ^2^Occupational Medicine Department; ^3^Anesthesia and Intensive Care Department, Sahloul Academic hospital, sousse, Tunisia

## Abstract

**Introduction:**

At the beginning of the Covid-19 pandemic, respiratory expression of SARS-CoV-2 infection was the most worrying one. Later, other symptoms appeared to be more disturbing such as neurological and psychiatric manifestations, which may be due to direct or indirect effects of this virus on the central nervous system.

**Objectives:**

To assess the prevalence of neuropsychological manifestations of covid-19 in healthcareworkers and to identify their risk factors.

**Methods:**

This is a cross-sectional descriptive epidemiological study, carried out in the teaching hospitals of Farhat Hached and Sahloul of Sousse. All healthcareworkers, having tested positive for SARS-COV 2 during the period from 01/09/2020 to 28/02/2021 were enrolled. The collection of socio-professional and medical data was based on a pre-established synoptic form completed during an interview with the participants.

**Results:**

A total of 953 COVID-19 patients were enrolled in this study. The mean age was 40.1 ± 10.5 years, with a sex ratio of 0.32. In our sample, 37.9% of patients had comorbidities such as psychiatric history (4.9%) and neurological history (2.4%). The prevalence of neuropsychological manifestations of covid-19 was 72.6%. The main neuropsychological manifestations were headache (50.3%), anosmia (40.7%), dysgeusia (29.9%), sleep disturbances (0.5%), dizziness (1, 2%) and paresthesia (0.3%).

Neuropsychological symptoms of long covid were dominated by memory impairment (10.7%), anosmia (8.5%), headache (7.3%), dizziness (3.4%) and sleep disturbances (3.1%).

The occurrence of neuropsychological manifestations was significantly associated with age (OR=1.6; p<10^-3^), male gender (OR= 0.57; p=0.03), smoking (OR=1.7; p=0.033), history of hypertension (OR=1.6; p=0.038), history of diabetes (OR=2.4; p<10-3) and hospitalization (OR=4.03 ; p<10^-3^).

**Conclusions:**

The high prevalence of neuropsychological manifestations underlines the importance of studying their pathogenesis in order to better adapt their therapeutic protocols.

**Disclosure of Interest:**

None Declared

